# EZH2 dependent H3K27me3 is involved in epigenetic silencing of ID4 in prostate cancer

**DOI:** 10.18632/oncotarget.2262

**Published:** 2014-07-25

**Authors:** Swathi Chinaranagari, Pankaj Sharma, Jaideep Chaudhary

**Affiliations:** ^1^ Center for Cancer Research and Therapeutic Development, Clark Atlanta University, Atlanta, GA

**Keywords:** ID4, EZH2, epigenetics, H3K27me3, prostate cancer

## Abstract

Inhibitor of DNA binding/differentiation protein 4 (ID4) is dominant negative helix loop helix transcriptional regulator is epigenetically silenced due to promoter hyper-methylation in many cancers including prostate. However, the underlying mechanism involved in epigenetic silencing of ID4 is not known. Here, we demonstrate that ID4 promoter methylation is initiated by EZH2 dependent tri-methylation of histone 3 at lysine 27 (H3K27me3). ID4 expressing (LNCaP) and non-expressing (DU145 and C81) prostate cancer cell lines were used to investigate EZH2, H3K27me3 and DNMT1 enrichment on ID4 promoter by Chromatin immuno-precipitation (ChIP). Enrichment of EZH2, H3K27Me3 and DNMT1 in DU145 and C81 cell lines compared to ID4 expressing LNCaP cell line. Knockdown of EZH2 in DU145 cell line led to re-expression of ID4 and decrease in enrichment of EZH2, H3K27Me3 and DNMT1 demonstrating that ID4 is regulated in an EZH2 dependent manner. ChIP data on prostate cancer tissue specimens and cell lines suggested EZH2 occupancy and H3K27Me3 marks on the ID4 promoter. Collectively, our data indicate a PRC2 dependent mechanism in ID4 promoter silencing in prostate cancer through recruitment of EZH2 and a corresponding increase in H3K27Me3. Increased EZH2 but decreased ID4 expression in prostate cancer strongly supports this model.

## INTRODUCTION

ID4 (Inhibitor of differentiation 4), a helix loop helix (HLH) protein is a dominant negative transcriptional regulator of basic HLH (bHLH) transcription factors [1]. ID4 is required for normal prostate morphogenesis where it is specifically expressed in the luminal epithelial cells [2]. Prostates from Id4−/− mice exhibit decreased branching morphogenesis and often display prostatic intra-epithelial neoplastic lesions (PIN) [2]. Loss of ID4 expression is also frequently observed in prostate cancer suggesting its essential role as a tumor suppressor [3, 4]. Knockdown of ID4 in LNCaP prostate cancer lines results in aggressive growth, increased cell survival and acquisition of castration resistance phenotype usually associated with advanced disease [5].

In addition to prostate cancer, decreased ID4 expression is also observed in leukemia [6], AML [7-11], CLL [12], ALL [13], glial neoplasia [14], squamous cell carcinoma [15], gastric cancer [16], pancreatic cancer [17], colorectal adenocarcinoma [18, 19], malignant lymphoma [20], cholangiocarcinoma [21], Barrett's esophagus and esophageal adenocarcinoma [22] and lung cancer [23].

ID4 gene as a whole, including 5′ and 3′ UTRs and the proximal promoter is rich in CpG islands (CGI) [16]. The CGI rich promoters also tend to be enriched for Sp1 and EBox response elements. In fact, Sp1 and E-box transcription factors within the CGIs around the TATA box are also required for ID4 transcriptional regulation [24]. Therefore accessibility of CG rich regulatory sites such as Sp1 and EBox within CGIs are dependent on CpG methylation. Indeed, in many of the cancers listed above including prostate, ID4 promoter is hyper-methylated around the TATA box. Thus epigenetic inactivation of ID4 due to promoter methylation appears to be the key mechanism in many cancers.

DNA methylation is a complex series of epigenetic re-programming events which involves precise interaction of a multitude of chromatin proteins including assembly of polycomb repressive complex 2 and 1. EZH2 (enhancer of Zeste 2) is part of the Polycomb repressor complex 2 (PRC2) that also includes SUZ12 (suppressor of Zeste 2) and EED (embryonic ectoderm development). Together, this PRC2 complex is involved in epigenetic re-programming in both normal and disease states including cancer [25]. As a histone methyl transferase, EZH2 is specifically involved in covalent modification of histone tails, specifically tri-methylation (me3) of lysine 27 (K27) on histone 3 (H3) (H3K27me3), a repressive mark found on many gene promoters that are silenced [26]. EZH2, as part of the PRC2 complex also recruits DNMTs (DNA methyl transferases) that in turn promotes DNA methylation at CpG islands (CGI) thus connecting the two key epigenetic repression systems [27]. EZH2 plays a critical role in cell fate determination and its increased expression is observed in many cancers [28], including prostate cancer [29, 30].

Collective observations led us to hypothesize that increased EZH2 expression could be involved in down-regulation of ID4 in prostate cancer. Here, we report, to our knowledge for the first time, that ID4 is a PcG (Polycomb group) protein EZH2 target gene in prostate cancer. Assembly of PRC2 complex on ID4 promotes repressive H3K27me3 histone modification and recruits DNMT1 resulting in ID4 promoter hyper-methylation.

## RESULTS

### Meta-Analysis of ID4 promoter methylation

**Figure 1:**
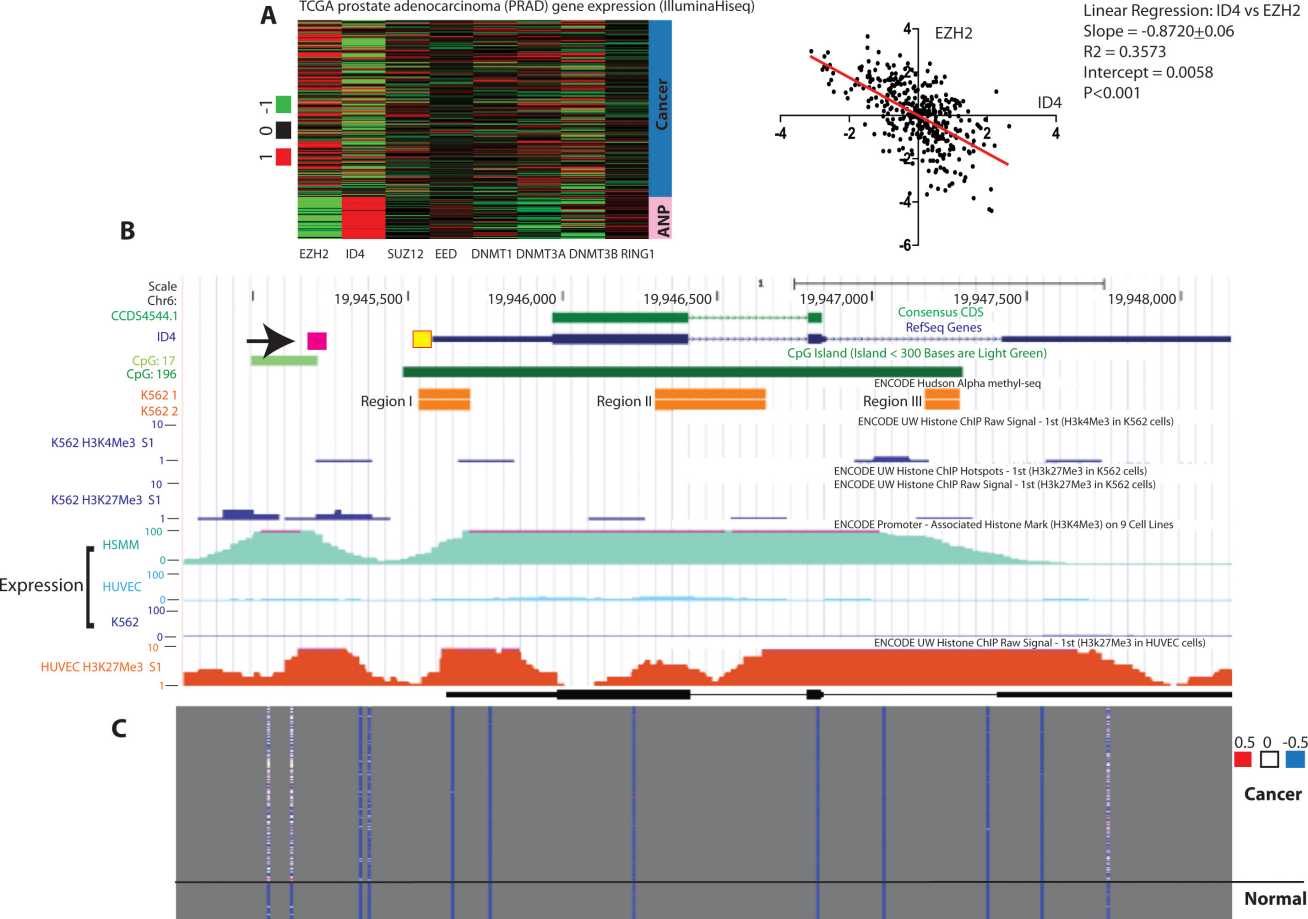
Meta-analysis of ID4 expression and epigenetic re-programming in prostate cancer and cell lines **A:** Right panel - The cancer genome Atlas (TCGA) prostate adenocarcinoma (PRAD) gene expression (IlluminaHiseq) data set was used to investigate expression of ID4 and genes involved in the formation of PRC2 and PRC1 complex. The cancer samples are represented by the blue bar and the adjacent normal by pink bar as indicated on the right (n=302). Left Panel – The EZH2 and ID4 normalized expression data for each sample in TCGA PRAD dataset (Right panel) was plotted to calculate the correlation between ID4 and EZH2 expression. The results of the statistical analysis are shown. A significant (P<0.001) inverse correlation (negative slope) was observed between EZH2 and ID4 expression **B:** The custom UCSC genome browser tracks showing location of ID4 gene (ID4), the protein coding region (CCDS), CpG islands (CpG:17 and CpG196), Methylated regions in K562 cell line (K562 1 and 2), H3k27Me3 marks in K562 and HUVEC (bottom, scale 1 to 10) and Expression of ID4 in HSMM, HUVEC and KG62 cell lines (scale 1 to 100). The source of each dataset is indicated above each track. The yellow box next to 5′UTR in ID4 ref-seq track (blue) is the location used for methylation specific PCR, whereas the 5′ pink box (indicated by arrow) is the region used to investigate EZH2, H3K27Me3, DNMT1 and H3Ac enrichment which corresponds to H3K27me3 marks in K562 and HUVEC tracks. C: ID4 promoter methylation profile extracted from TCGA prostate adenocarcinoma (PRAD) DNA methylation (HumanMetylation450) dataset (see above). The degree of methylation is indicated by blue (un-methylated) and red (Hyper-methylated) in relation to ID4 gene (top panel).

To address the mechanism involved in the epigenetic regulation of ID4, we first analyzed the expression of genes involved in the DNA methylation including DNMTs (DNMT1, DNMT3A and DNMT3B) and constituents of PRC2 (EZH2, Suz12, EED) and PRC1 (RING1) complex in The Cancer Genome Atlas (TCGA) [31] prostate cancer adenocarcinoma (PRAD) gene expression (IlluminaHiseq) database (Fig. 1A). The meta analysis suggested high ID4 expression in adjacent normal samples was associated with low EZH2 whereas inverse was observed in cancer samples i.e. low ID4 but high EZH2 expression (Fig. 1A, right panel). The corresponding normalized EZH2 and ID4 expression values for each sample were obtained from the TCGA dataset and plotted to obtain a correlation between ID4 and EZH2 (Fig. 1A, left panel). A significant (P<0.001) negative correlation (slope -0.8720+0.06) and correlation coefficient R2 = 0.3573 suggested that ID4 expression is inversely related with EZH2 expression. These results were used to develop the hypothesis that EZH2 could be involved in epigenetic silencing of ID4 in prostate cancer. EZH2 forms a multimeric protein complexes e.g. with EED and catalysis tri-methylation (Me3) of lysine 27 (K27) of histone 3 (H3). We next explored the embedded “expression” and “regulation” tracks using UCSC genome browser to further explore if in fact histone modifications such as EZH2 mediated H3K27Me3 is observed on ID4 promoter in cells that lack ID4 expression such as chronic myelogenous leukemia cell line K562 [32].

Consistent with earlier reports, the first predicted CpG island (proximal) in ID4 gene in UCSC genome browser was observed essentially spanning the entire length of the gene (CpG:196 track, Fig1B), including proximal promoter whereas the second CpG island was approximately 400bp upstream of the transcriptional start site (CpG:17 track, Fig. 1B).

Two non-ID4 expressing cell lines HUVEC and K562 [32] and ID4 expressing cell line HSMM (Human Skeletal Muscle Myoblasts) were used to investigate histone modifications and respective promoter methylation in USCS genome browser. The rationale behind this approach was to consolidate our hypothesis that histone modification, in part due to EZH2 recruitment is involved in ID4 gene silencing. Active methylation in K562 was observed (ENCODE Hudson Alpha Methyl-seq, similar dataset for HUVEC was not available) around the proximal promoter which was consistent with the studies reported earlier in other cell lines/ tissues [32]. The CpG methylation marks in K562 cell lines was associated with increased H3K27Me3 marks (repressive) but no H3K4Me3 marks (active) were observed. Similar repressive H3K27Me3 marks were observed in the HUVEC cell lines. The HSMM (Human Skeletal Muscle Myoblasts) cells expressed ID4 but the methylation and Histone modification data were not available in the USCS genome browser at the time of this study. These results strongly suggested that H3K27Me3 could be associated with ID4 epigenetic inactivation supporting the relevance of EZH2 in this process. ID4 promoter was also methylated in prostate cancer but un-methylated in adjacent normal in TCGA datasets (Fig. 1C) that is supported by methylation specific PCR in tissue samples as reported earlier [3]. Collectively, data mining supported EZH2 dependent histone modification, specifically H3K27me3 on ID4 promoter.

### ID4 and EZH2 expression in prostate cancer cell lines

**Figure 2:**
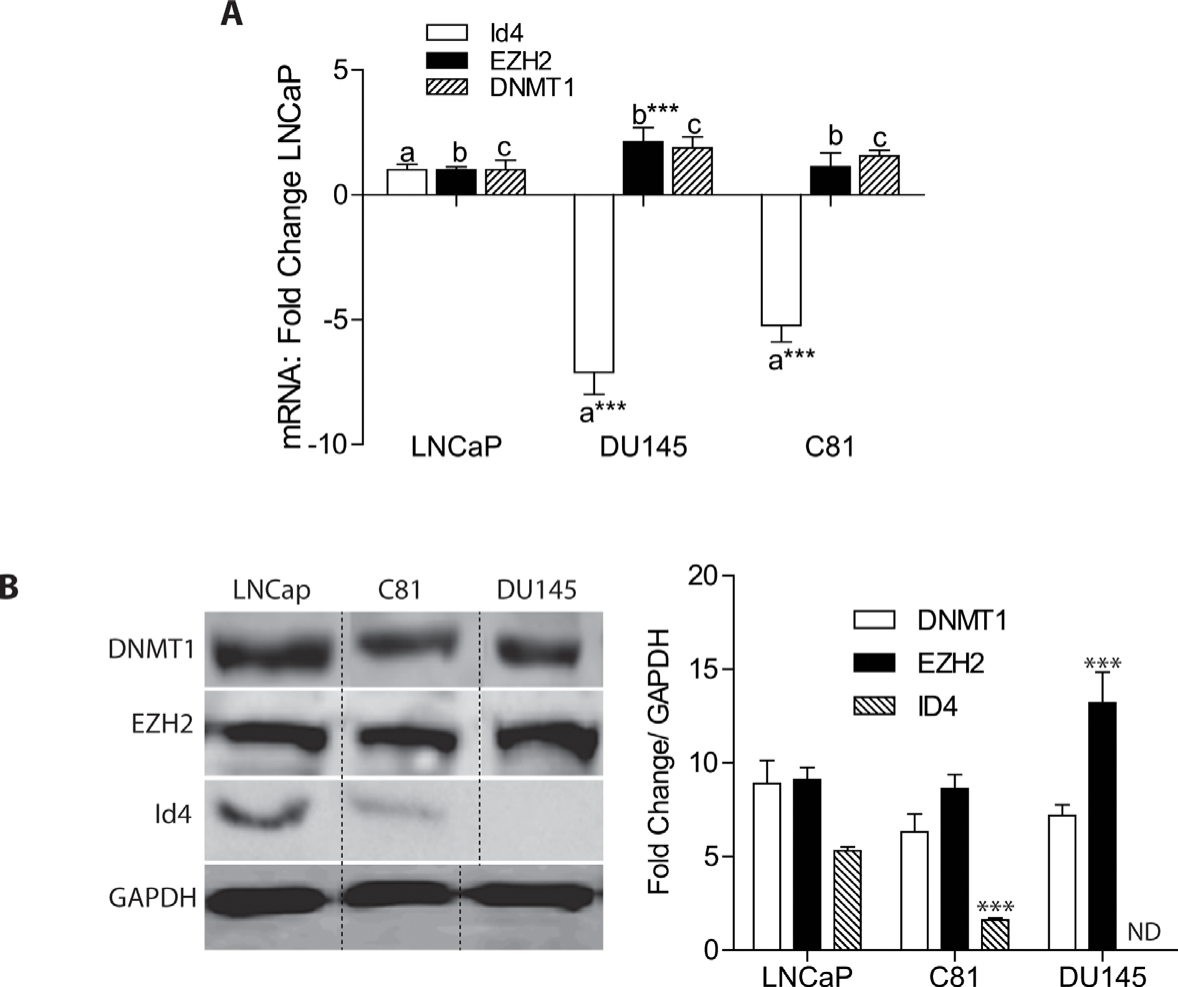
Expression of ID4, EZH2 and DNMT1 in prostate cancer cell lines **A:** Quantitative real time RT-PCR of respective genes. The data (mean+ SEM, n=3 in triplicate) is normalized to GAPDH followed by fold change over LNCaP cells. The bars marked with “a”, “b” and “c” corresponding to ID4, EZH2 and DNMT1 demonstrate statistical differences with LNCaP cells (***: P<0.001). **B:** Right panel - Western blot analysis of DNMT1, EZH2 and ID4 expression in LNCaP, DU145 and C81 cells. GAPDH was used as loading control. Representative data of 3 independent experiments is shown. Left Panel – Semi-quantitative analysis of the western blot data (right panel). The data is normalized to GAPDH (mean+SEM, n=3, ***: P<0.001 as compared to LNCaP cells).

Our previous studies demonstrated that ID4 promoter is hypo-methylated in LNCaP cells but hyper-methylated in DU145 cells [3]. The C-81 cells, a more metastatic, androgen insensitive derivatives of LNCaP cells also demonstrate ID4 promoter hyper-methylation [3]. These three cells lines were used as models to understand the mechanism by which ID4 promoter is epigenetically regulated. Consistent with promoter methylation status, significantly lower ID4 transcript (Fig. 2A) and protein (Fig. 2B) was observed in DU145 and C-81 cells as compared to LNCaP cells. The EZH2 transcript and protein expression was significantly higher in DU145 cells but no change was observed in C81 cells as compared to LNCaP cells (Fig. 2A and B). These results are also consistent with other studies showing that EZH2 transcript and protein expression is higher in DU145 cells as compared to LNCaP [33, 34]. DNMT1 was also expressed in all three cell lines at similar levels. These results suggested that ID4 promoter hyper-methylation in DU145 and C-81 and hypo-methylation in LNCaP is not dependent on EZH2 and DNMT1 expression which prompted us to investigate whether these proteins are actively recruited on the ID4 promoter leading to differential methylation patterns.

### ID4 promoter is enriched with EZH2 and H3K27me3 in DU145 and C81 cells

The promoter regions showing higher H3K27me3 marks in K562 and HUVEC cells (Fig. 1B) were used to investigate whether these regions are also enriched for EZH2 and H3K27me3 in prostate cancer cells. We designed primers to scan +38 to -400 bp region of the ID4 promoter to investigate EZH2 and/or H3K27me3 enrichment. This area was selected based on the USCS genome browser dataset (Fig. 1). The primer set spanning -346 to -238bp upstream of the transcriptional start site yielded a positive signal EZH2 and H3K27me3 enrichment. This region also corresponds to H3K27me3 enrichment in HUVEC and K462 cell lines (Fig. 1B).

**Figure 3:**
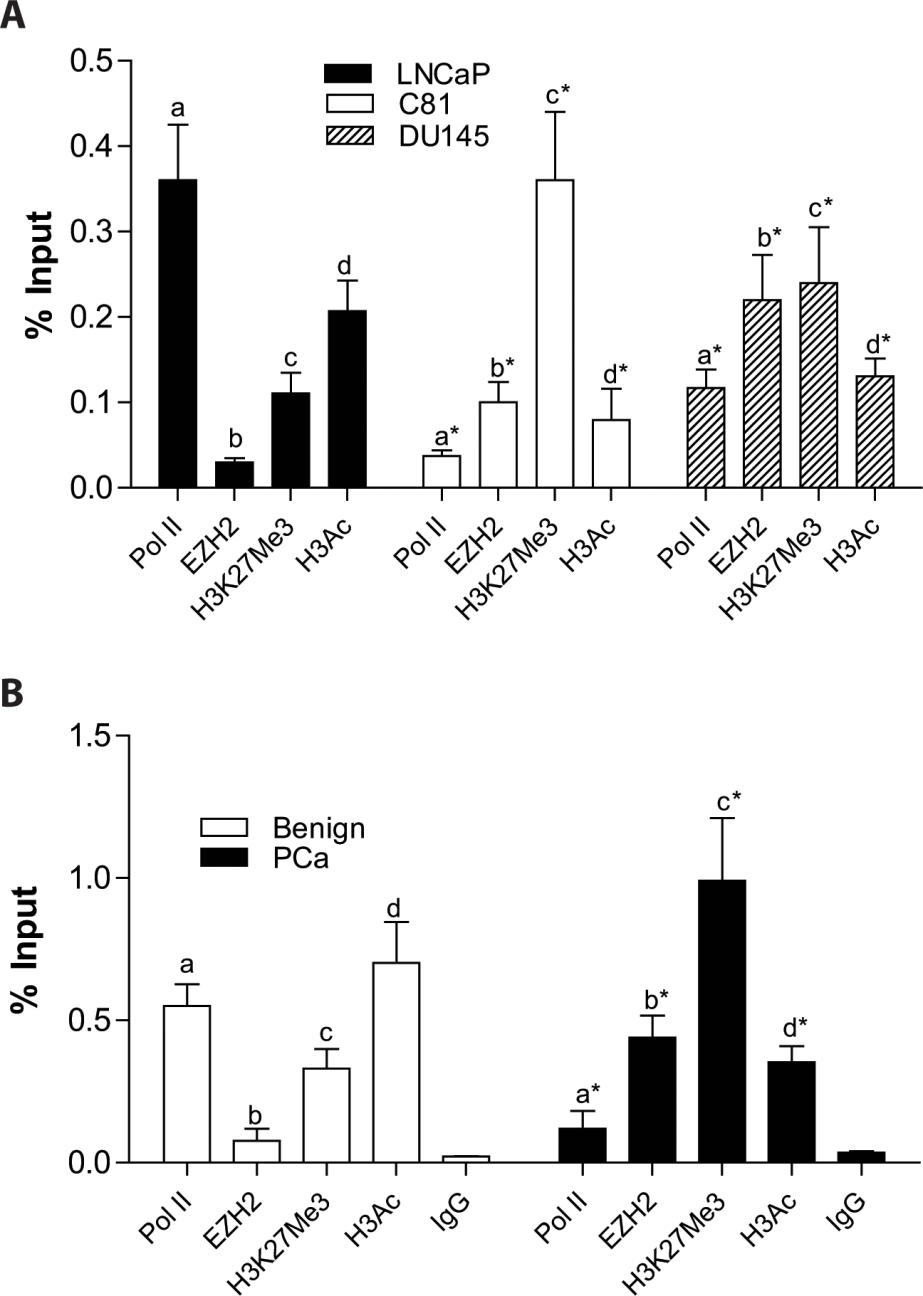
Enrichment of EZH2 and histone modifications on ID4 promoter in prostate cancer cell lines and tissue on Chromatin immuno-precipitated (ChIP) DNA **A:** Enrichment of Pol II, EZH2, H3K27me3 and H3Ac on ID4 promoter in prostate cancer cell lines LNCaP, DU145 and C81 cells. The data is expressed (mean+SEM, n=3 in triplicate) as % of input. The statistical significance between enrichment (indicated by letters “a”, “b”, “c” and “d” corresponding to Pol II, EZH2, H3K27me3 and H3Ac respectively) is based on comparison with LNCaP cells (*: P<0.001). **B:** Similar to “A” above but the enrichment was performed on DNA isolated from FFPE cancer/ normal prostate tissue by laser capture micro-dissection. The number of Benign and prostate cancer samples were 5 each.

Chromatin immuno-precipitation experiments demonstrated that ID4 promoter is enriched with EZH2 in C81 (% input 0.11+0.024, P<0.001) and DU145 (0.23+0.0.052, P<0.001) cells as compared to LNCaP cells (EZH2: 0.03+0.004). The corresponding H3K27me3 enrichment was also higher in DU145 and C81 cells (0.36+0.084 and 0.24+0.065 in C81 and DU145 respectively) as compared to LNCaP cells (0.11+0.021). Decreased RNA polymerase II (PolA) occupancy in C81 (0.037+0.0054) and DU145 (0.116+0.022) as compared to LNCaP (0.36+0.061) is also consistent with ID4 transcription (Fig. 3A) in these three cell lines. Actively transcribed genes also commonly exhibit increased H3 acetylation as opposed to decreased H3K27me3. As expected, decreased H3 acetylation was observed in C81 (0.079+0.036, P<0.001) and DU145 (0.13+0.021, P<0.001) cells as compared to LNCaP cells (0.26+0.033) (Fig. 3A).

Laser capture micro-dissected prostate cancer and benign prostate tissue was used to further investigate EZH2 dependent histone modifications. The prostate cancer samples (n=5) and benign prostate tissue (n=5) were drawn from our well established sample set with validated ID4 promoter methylation status [3]. EZH2 enrichment in benign prostate tissue was significantly lower (0.076+0.042) as compared to that in prostate cancer (0.44+0.077, P<0.001) (Fig. 3B). H3K27me3 enrichment also revealed a similar profile, i.e. low in benign (0.31+0.071) and high enrichment in prostate cancer tissue (0.99+0.26, P<0.001). In contrast, ID4 promoter was highly enriched for the RNA polymerase II and H3 acetylation (0.55+0.078 and 0.70+0.144, P<0.001, respectively) in benign prostate as compared to prostate cancer (0.12+0.061 and 0.35+0.051, respectively) (Fig. 3B). Together, results from prostate cancer cell lines and prostate tissue suggested that ID4 is regulated in part by histone modifications in an EZH2 dependent manner.

### Knockdown of EZH2 leads to re-expression of ID4

**Figure 4:**
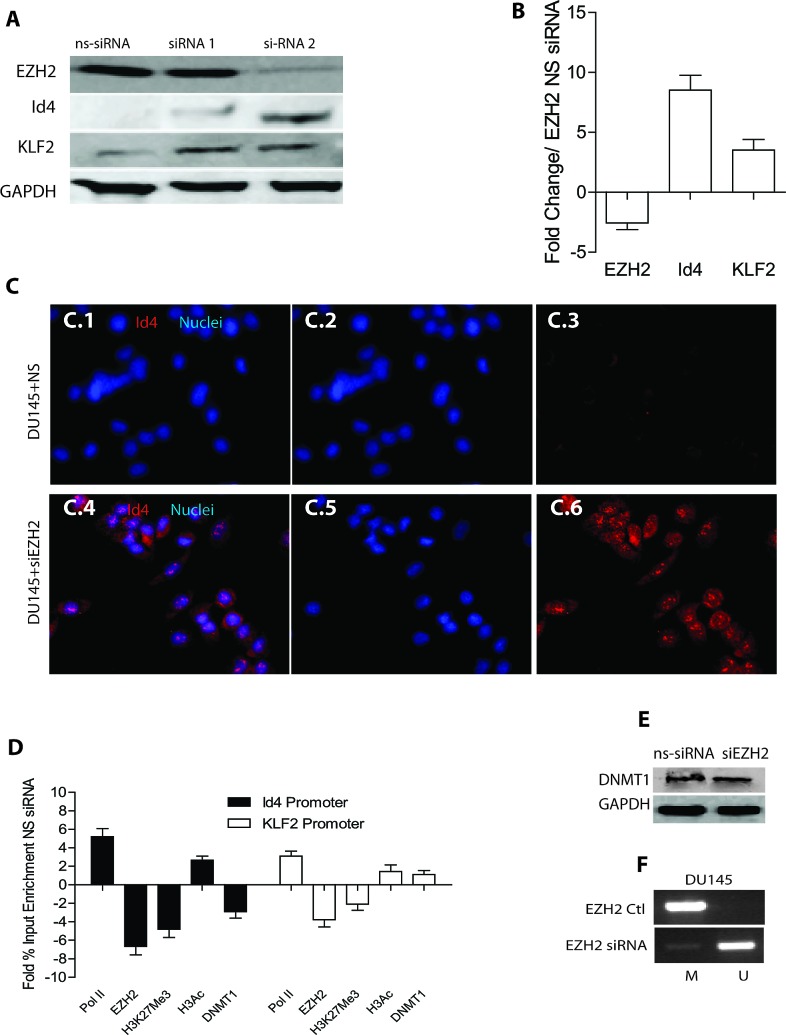
Effect of EZH2 silencing on ID4 expression **A:** Two different siRNAs (siRNA1 and siRNA2) were used to transiently knock down EZH2 in DU145 cells followed by western blot analysis of EZH2, ID4 and KLF2 (used as a positive control for EZH2 dependent down-regulated gene). Increase in ID4 expression with greater EZH2 knock down was observed with siRNA2 that was used for all subsequent studies. Representative western blot is shown. **B:** Real time quantitative RT-PCR analysis of corresponding gene expression following EZH2 knockdown by siRNA2. The data is expressed (mean+SEM, n=3 in triplicate) as fold change compared to non-specific siRNA. **C:** Immuno-cytochemical analysis of ID4 expression following siRNA2 mediated knockdown of EZH2 in DU145 cells (x200 magnification). ID4 expression is in red and the nuclei in Blue (DAPI). C.1 and C.4 are merged images of Blue (Nuclei, C.2 and C.5) and Red (ID4, C.3 and C.6). C.1, C.2 and C.3 are DU145 cells transfected with non-specific siRNA (DU145+NS). Panels C.4, C.5 and C.6 are DU145 cells transfected with EZH2 siRNA (DU145+siEZH2). Representative images are shown. D: Enrichment of EZH2, H3K27me3, H3Ac and DNMT1 on ID4 and KLF2 promoters following EZH2 knockdown in DU145 cells. The data is expressed (mean+SEM, n=3 in triplicate) fold change of % input as compared to DU145 cells transfected with non-specific EZH2 siRNA. E: Western blot analysis of DNMT1 expression in DU145 cells with non-silencing siRNA or with EZH2 si-RNA2 (siEZH2). Representative of 3 blots is shown. F: Methylation specific PCR (MSP) on ID4 promoter following knockdown of EZH2 in DU145 cells. A band in “M” lane represents methylation of ID4 promoter where as a band in “U” lane represents un-methylated promoter. Representative results are shown.

To determine whether EZH2 in fact down-regulates ID4 expression directly, we performed RNA interference mediated knockdown of EZH2 in DU145 cells (DU145+siEZH2). The immuno blot analysis in DU145 cells showed knockdown of EZH2 with siRNA2 to a greater extent as compared to siRNA1 and non-specific siRNA (ns-siRNA) 72h after transfection (Fig. 4A). Based on these results, all subsequent studies were performed with EZH2 siRNA2. The results from quantitative real time PCR revealed that a significant increase in the expression of ID4 and KLF2 (Fig. 4B) in DU145+siEZH2 cells as compared to non-silencing controls (DU145+siNS). KLF2 was used as a positive control which is down-regulated by EZH2 [35]. The immuno-fluorescence analysis also revealed increased ID4 expression in DU145+siEZH2 as compared to non-silencing controls (Fig. 4C4). Knockdown of EZH2 also resulted in decreased EZH2 specific H3k27me3 repressive marks with a corresponding increase in the enrichment of transcriptionally active H3 acetylation marks and RNA polymerase II on ID4 promoter (Fig. 4D).

EZH2 physically interacts with and recruits DNA methyl-transferases DNMT1, 3A and 3B to promote methylation and establish stable repressive chromatin structures [27] suggesting that histone modifications acts upstream of methylation and/or its initiation. Previous studies form our laboratory have shown that treatment of DU145 cells with 5-azacitidine leads to re-expression of ID4 [36]. 5-azacitidine promotes proteosomal degradation specifically of DNMT1 [37] suggesting that ID4 promoter hyper-methylation in DU145 cells is in part mediated by DNMT1. Based on these studies, we next investigated whether DNMT1 is also recruited on ID4 promoter in an EZH2 dependent manner. Results from Chromatin immuno precipitation experiments suggested significantly decreased DNMT1 enrichment on ID4 promoter in DU145+siEZH2 as compared to DU145+siNS cells (Fig. 4D). Surprisingly, enrichment of DNMT1 on KLF2 promoter was not significantly different between DU145+siEZH2 and DU145+siNS cells. KLF2 promoter is also an EZH2 target gene in many cancers [35] and recently shown to be hyper-methylated by DNMT1 in endothelial cells [38]. The reduction in DNMT1 enrichment on ID4 promoter could be due to its decreased expression following EZH2 knockdown. In order to confirm this, we investigated the expression of DNMT1 in DU145+siEZH2 and DU145+siNS cells (Fig. 4E). Surprisingly, the DNMT1 levels were similar between DU145+siEZH2 and DU145+siNS cells. These results suggested that the reduced DNMT1 recruitment on ID4 promoter was due to decreased EZH2 recruitment and not due to decreased expression of DNMT1. Next, we investigated whether increased ID4 expression in DU145+siEZH2 (Fig.4A and 4C) was due to decreased ID4 promoter hyper-methylation. The methylation specific PCR on ID4 promoter using bisulfite treated DNA from DU145+siEZH2 cells reveled decreased promoter methylation as compared to DU145+siNS (Fug. 4F). Together, these results suggested that EZH2 silencing leads to decreased DNMT1 recruitment resulting in ID4 promoter hypo-methylation.

### Knockdown of EZH2 results in Hypo-methylation of ID4 promoter

**Figure 5:**
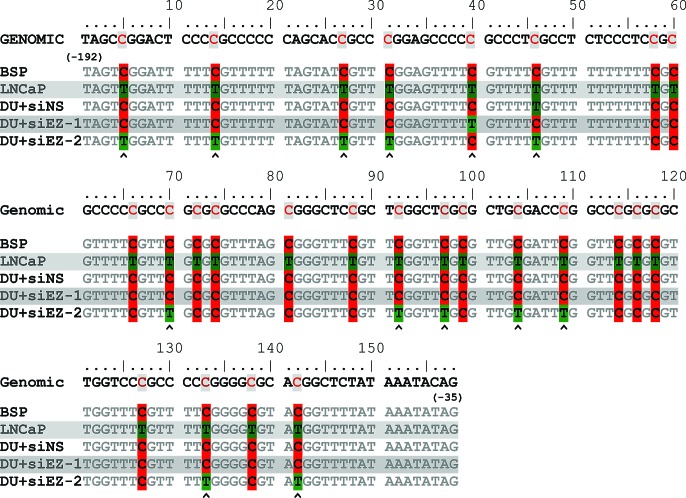
Bisulfite sequence of MSP/ USP region of ID4 promoter The Genomic sequence is indicated at the top (-192 to -35 bp from transcriptional start site). BSP: The predicted sequence after bisulfite conversion. The consensus sequences from LNCaP, DU145+siNS (DU+siNS), DU145+siEZH2 (DU+siEZ) are represented. The methylated Cytosine (C, red) and un-methylated Cytosine converted to Thymidine (T, Green) after bisulfite conversion are indicated. Polymorphism was observed at CpG islands in DU145+siEZH2 (C/T) hence two sequences are displayed. The read from two representative sequences is shown. The arrow heads at the bottom indicates possible site of hypo-methylation on ID4 promoter in DU145+siEZH2 cells.

Finally, direct bisulfite sequencing was performed on DU145+siNS, DU145+siEZH2 and LNCaP cells. The sequence of the region amplified MSP primers (the sequencing primers flanked the MSP region shown in Fig. 4F) confirmed, as expected that the CpG islands in LNCaP cells were hypo-methylated (conversion of “C” to “T” by bisulfite reaction) but were hyper-methylated in DU145+siNS (no conversion of “C” due to methylation). Partial conversion of “C” to ”T” was observed in DSU145+siEZH2 cells. Sequence alignments allowed us identify critical CpG islands (indicated by arrow heads (Fig. 5) that were hypo-methylated in DU145+siEZH2 cells resulting in ID4 expression (Fig. 4). These results led us to conclude that EZH2 recruitment promotes ID4 hypermethylation through a complex process involving H3K27me3 and DNMT1.

## DISCUSSION

The data presented here supports an EZH2 dependent epigenetic silencing of ID4 in prostate cancer. This conclusion is particularly compelling when the experimental data (cell line and clinical studies) is compared to the meta-analysis of ID4 promoter (UCSC genome browser) and remarkable mutually exclusive expression profile of EZH2 and ID4 in prostate cancer (TCGA datasets and experimental evidence).

A number of studies support the role of EZH2 as an oncogene in prostate cancer that is typically associated with increased risk of metastasis and recurrence [29, 30, 39]. Knockdown of EZH2 in prostate cancer cell lines results in decreased cellular growth and invasion [34, 40-42]. EZH2 mediated transcriptional repression of putative tumor suppressors such as E-cadherin [43] via increased H3K27me3 is dependent on SET domain that in addition to methyl transferase activity also requires histone de-acetylase activity, possibly through recruitment of HDAC2 by EZH2, itself a component of PRC2 complex [44]. Such a co-operative histone modification is clearly observed on ID4 promoter where increased EZH2 dependent histone methylation is associated with decreased histone acetylation, further contributing to the repressive histone marks.

ID4 expression is also tightly controlled by epigenetic mechanisms during oligodendrocyte differentiation by PRMT5, a type II protein arginine methyltransferase. PRMT5 associates with ID4 CpG islands and is required for maintaining its methylation status and subsequent gene silencing in differentiating oligodendrocyte [45]. In prostate cancer cells PRMT5 expression is primarily cytoplasmic and promotes growth. In contrast, PRMT5 is nuclear in benign prostate epithelial cells where it inhibits growth [46]. Thus PRMT5 localization (predominantly cytoplasmic) in prostate cancer does not correspond with its role in ID4 methylation or association with CpG islands, which as one would expect to be in the nucleus. However direct evidence demonstrating the ID4 gene expression is independent of PRMT5 in prostate cancer remains to be investigated.

Re-expression of ID4 by silencing EZH2 suggests that EZH2 dependent H3K27me3 could be an early event in establishing this histone code that may recruit DNA methyl- transferases to promote DNA methylation. We and other have demonstrated that inhibition of DNMT1 by 5- Azacitidine treatment also promotes ID4 expression in DU145 [4, 36], clearly suggesting that these two processes are inter-related. Indeed, studies have shown that treatment of cells with 5-Aza results in removal of H3K27me3 marks without altering the expression of EZH2 or other Histone methyl transferases [47]. Furthermore, we observed that methyl transferases such as EZH2 were present in the same region as DNMT1 on ID4 promoter, possibly in the same protein complex [27, 48, 49]. Thus interfering with either EZH2 (siRNA) or DNMT1 (5-Aza) could de-stabilize the epigenetic mark resulting in increased ID4 expression. Thus increased EZH2 expression and its subsequent recruitment appears to be the primary mechanism involved in epigenetic silencing of ID4 in prostate cancer. The role of other co-operating proteins within PRC1 and PRC2 cannot be ruled out as their expression/ recruitment could alter ID4 methylation/ histone modifications. Lack of a significant change in the expression of DNMT1 following EZH2 silencing further suggests that recruitment of protein complexes takes precedence over expression in epigenetic modifications at least in context of ID4. This is partly reflected in the TCGA expression profile where the expression of other PRC1/2 complex proteins does not change to the extent as compared to EZH2 in normal prostate and prostate cancer. Evidence that assembly and not expression of PRC1/2 complex proteins is dependent on recruitment EZH2 as the initial step is also evident from studies indicating that the expression of BM1-1, SIRT-1, DNMT1 and DNMT3B is not associated with prostate cancer [50].

Whether the EZH2-DNMT mechanism is specific to prostate cancer or a more general pro-cancer pathway involved in ID4 gene silencing remains to be investigated. It is also possible that ID4 gene regulation may be distinct in cancer cells versus cell undergoing proliferation/ differentiation that require stage specific accessibility to ID4 transcriptional regulators such as those involving sp1/ bHLH/ hormones through alternate mechanism e.g PRMT5 cellular localization.

The EZH2-DNMT dependent mechanism at least in prostate cancer suggest that targeting this pathway through specific inhibitors resulting in general epigenetic re-programming including up-regulation of ID4 could be a strong therapeutic strategy. Our previous studies have shown that ectopic ID4 expression alone results in cell cycle arrest [36], induction of apoptosis and senescence [51], activation of p53 [52] and increased sensitivity to chemo-therapeutics [51]. Thus strategies that can either specifically re-program ID4 promoter or target ID4 dependent downstream pathways are strong therapeutic approaches that needs to be explored.

## MATERIALS AND METHODS

### Prostate cancer cell lines

LNCaP and DU145 prostate cancer cell lines were purchased from ATCC and cultured as per ATCC recommendations and as described previously [3]. C-81 cells were kindly provided by Prof. Ming-Fong Lin (Dept. Biochemistry and Molecular Biology and Eppley Institute for Cancer, University of Nebraska Medical Center, Omaha, NE) [53].

### EZH2 siRNA transient transfections

Cells were seeded in 6 well plates at 1.25×10^5^ cells/well in 5% Bovine Calf Serum (VWR) and allowed to attach overnight. The following day, cells were rinsed with serum free media immediately prior to transfection. Transient transfection reagent (Santa Cruz) was used to transfect EZH2 siRNA (cell signaling, cat # 6509S (siRNA1) or Santa Cruz, cat # SC-35312 (siRNA2)) and EZH2 siRNA control (Santa Cruz, cat # SC-37007) in DU145 cells according to manufacturer's protocol and the cells were then cultured for additional 48-72 hours. Subsequently, the transiently transfected cells were harvested for RNA/protein or cross linked with formaldehyde for Chromatin immuno-precipitation.

### Prostate Tissue Samples

Duplicate ten micron FFPE (Formalin fixed and paraffin embedded) sections from age matched prostate cancer (mean age 64.3+ 2.4) and benign prostate hyperplasia (mean age 61.8 + 3.1) affixed on Leica PEN (polyethylene naphthalate) membrane coated slides were obtained from Cooperative Human Tissue Network (CHTN), Southern Division at University of Alabama at Birmingham following appropriate IRB approvals. Specific cancerous and non-cancerous regions were isolated by Laser Capture micro-dissection for subsequent analysis as described previously [3].

### Chromatin Immuno-precipitation (ChIP) Assay

Formalin-fixed paraffin-embedded (FFPE) samples (see above) were used for ChIP based analysis for enrichment of EZH2, H3K27me3 and H3Ac on ID4 and KLF2 promoter. For this analysis, the regions showing >75% cancerous regions or more than >80% normal/ benign regions were dissected using Leica LMD6500 and captured in micro centrifuge tubes. Genomic DNA was isolated from these sections by the method of Fanelli et al., [54] except that tissue samples were de-paraffinized with xylene instead of histolemon. The chromatin extracted from tissue samples was sheared (Covaris S220), subjected to immuno-precipitation with either EZH2 (Active Motif)), DNMT1 (Epigentek), RNA POL II (Millipore), H3Acetylation (global, Active Motif) or IgG (Millipore) antibodies, reverse cross linked and subjected to quantitative ChIP- PCR (qChIP). The qChIP was performed on sheared DNA with following primers: KLF2 [35] FP 5′- GAG ACT CCA GAC TTC CCA TCC; RP: 5′- CAG AGA CTC TCA GGG GAG CAC and ID4 FP: 5′- GAC TCC CAC TCA GCT CTC TT, RP: 5′- TGG AGT GGC CAG CCA ATC A. The ID4 primers amplified -346 to -238 bp region upstream of the transcriptional start site.

Chromatin immuno-precipitation in cell lines was performed using the ChIP assay kit (Millipore, Billerica, MD) as per manufacturer's instructions. The chromatin (total DNA) extracted from cells was sheared (Covaris S220), subjected to immuno-precipitation with respective antibodies (see above), reverse cross linked and subjected to quantitative ChIP- PCR in Bio-Rad CFX.

### Western Blot Analysis

30ug of total protein, extracted from cultured prostate cancer cell lines using M-PER (Thermo Scientific) was size fractionated on 4-20% SDS-polyacrylamide gel. The SDS-gel was subsequently blotted onto a nitrocellulose membrane (Whatman) and subjected to western blot analysis using respective protein specific antibodies: EZH2 (Cell signaling), ID4 (Bio-Check), KLF2 (Millipore), and GAPDH (Cell signaling). After washing with 1x PBS, 0.5% Tween 20, the membranes were incubated with *horseradish peroxidase (HRP)* coupled secondary antibody against rabbit IgG and visualized using the Super Signal West Dura Extended Duration Substrate (Thermo Scientific) on Fuji Film LAS-3000 Imager.

### RNA preparation, RT-PCR and quantitative RT-PCR (qRT-PCR)

Total RNA was extracted using TRIzol (Invitrogen, Carlsbad, CA) as described previously [55]. The reverse transcribed [36] RNA was used to perform qRT-PCR as described previously using gene specific primers [56]: *ID4:* Forward Primer (FP) 5′- TGC AGT GCG ATA TGA ACG AC, reverse primer (RP) 5′- AGC TGC AGG TCC AGG ATG TA- 3′; *EZH2:* FP 5′- TCG GTG ACC AGT GAC TTG GA, RP 5′- CTG CTG TAG GGG AGA CCA AGA; *DNMT1*: FP 5′- AAC CTT CAC CTA GCC CCA G, RP 5′- CTC ATC CGA TTT GGC TCT T; *KLF2*: FP: 5′- CGT CCT TCT CCA CTT TCG CCA G, RP 5′- GAA GTC CAG CAC GCT GTT GAG G; *GAPDH*: FP 5′- GAA GGT GAA GGT CGG AGT C, RP: 5′- GAA GAT GGT GAT GGG ATT TC.

### Immuno-cytochemistry

Cells were grown on glass chamber slides up to 75% confluency. The slides were then washed with PBS (3x) and fixed in ice cold methanol for 10 min at room temperature and stored at -20C until further use. Before use, the slides were equilibrated at room temperature, washed with PBS (5min x3), blocked with 1%BSA in PBST for 30 min at room temp and Incubated overnight (4C) with primary antibody (1% BSA in PBST, Table A.1). The slides were then washed in PBS and incubated with secondary antibody with fluorochrome conjugated to DyLight (Thermo Scientific) in 1% BSA for 1hr at room temp in dark. The slides were subsequently washed again and stained in DAPI (1μg/ml) for 1 min and mounted with glycerol. Images were acquired by Zeiss fluorescence microscope through Axiovision software.

### DNA Methylation Analysis

ID4 promoter methylation was analyzed using methylation-specific PCR (MSP) as described previously [3, 36, 57]. The MSP region amplified in context of the ID4 genome in this study has been previously investigated and well characterized in gastric [16], breast [57, 58] and colorectal cancers [59].

### Bisulfite Sequencing

Direct bisulfite sequencing of the PCR product was performed as reported earlier [16] using ABI sequencer.

### Statistical Analysis

Quantitative real time data was analyzed using the ΔΔCt method. The ChIP data was analyzed against non-immune IgG used as a negative control. Within group Student's t-test was used for evaluating the statistical differences between groups. Normalized expression values were extracted from TCGA PRAD dataset and were subjected to linear regression analysis in GraphPad Prism 5 to obtain correlation coefficient between EZH2 and ID4 expression in adjacent normal prostate and prostate cancer.
